# StrongestPath: a Cytoscape application for protein–protein interaction analysis

**DOI:** 10.1186/s12859-021-04230-4

**Published:** 2021-06-29

**Authors:** Zaynab Mousavian, Mehran Khodabandeh, Ali Sharifi-Zarchi, Alireza Nadafian, Alireza Mahmoudi

**Affiliations:** 1 Department of Computer Science, School of Mathematics, Statistics and Computer Science, College of Science, University of Tehran, Tehran, Iran; 2 School of Computing Science, Simon Fraser University, Burnaby, BC Canada; 3 Department of Computer Engineering, Sharif University of Technology, Tehran, Iran; 4 Department of Stem cells and Developmental Biology at the Cell Science Research Center, Royan Institute for Stem Cell Biology and Technology, ACECR, Tehran, Iran

**Keywords:** Protein–protein interaction network, Signaling network, Pathway reconstruction, Regulatory pathway, Cytoscape App

## Abstract

**Background:**

StrongestPath is a Cytoscape 3 application that enables the analysis of interactions between two proteins or groups of proteins in a collection of protein–protein interaction (PPI) network or signaling network databases. When there are different levels of confidence over the interactions, the application is able to process them and identify the cascade of interactions with the highest total confidence score. Given a set of proteins, StrongestPath can extract a set of possible interactions between the input proteins, and expand the network by adding new proteins that have the most interactions with highest total confidence to the current network of proteins. The application can also identify any activating or inhibitory regulatory paths between two distinct sets of transcription factors and target genes. This application can be used on the built-in human and mouse PPI or signaling databases, or any user-provided database for some organism.

**Results:**

Our results on 12 signaling pathways from the NetPath database demonstrate that the application can be used for indicating proteins which may play significant roles in a pathway by finding the strongest path(s) in the PPI or signaling network.

**Conclusion:**

Easy access to multiple public large databases, generating output in a short time, addressing some key challenges in one platform, and providing a user-friendly graphical interface make StrongestPath an extremely useful application.

## Background

The teamwork of proteins, in terms of temporary or permanent interactions, is critical for any biological process. There have been numerous protein–protein interaction (PPI) or signaling pathways databases developed based on the experimental approaches or computational predictions. Some of the databases assign different confidence levels to the interactions, e.g. higher confidences for the experimentally validated interactions, and lower values for the computationally predicted ones. So, the identification of the cascades of interactions from the receptors to the transcriptional regulatory factors is a major challenge in systems biology. To make this process easier, Cytoscape [1, 2] was developed to help with molecular and network profiling analysis and also visualizing molecular interaction networks. Other Plugins and Apps can be integrated into this flexible platform for complex network analysis and visualisations. PesCa [3], PathExplorer (http://apps.cytoscape.org/apps/pathexplorer) and PathLinker [4, 5], are examples of such apps that can compute paths in biological networks.

We developed StrongestPath, a Cytoscape 3.0 App, to address three key challenges during analysis of PPI or signaling networks. The first challenge is identifying a cascade of interactions, as a regulatory or signaling pathway, in a large PPI or signaling network. In many experimental studies perturbation of a protein *A* is observed to influence a protein *B*, but the cascade of interactions between *A* and *B* is unidentified. The second challenge addressed by StrongestPath is growing the sub-network of the input proteins, either by extracting their pairwise interactions from a list of PPI or signaling databases, or adding further proteins that are more likely to create protein complexes or dense interactions with the input proteins. To address this challenge, StrongestPath looks at the whole PPI or signaling network and identifies proteins with maximum total confidence of interactions with the given set of proteins. This feature can be used to identify unknown elements of a protein complex, biological process or core regulatory circuitry. The third challenge is identifying any activating or inhibitory regulatory path between two distinct groups of proteins. For example, when a list of genes is identified in a study of a phenomenon, researchers seek to answer whether there is a regulatory pathway between the transcription factors associated with the phenomenon and the identified genes regarding the experimentally validated data reported in the public databases [6].

StrongestPath comes with two types of built-in databases: (I) some PPI and signaling networks of human and mouse, containing interactions recorded in public databases, (II) protein nomenclature database, containing 11 different symbols and accession IDs of genes and proteins in different databases. In addition, users can provide their own networks and nomenclature datasets. This allows StrongestPath to be used for any organism, PPI, gene regulatory networks, and signal transduction networks.

Our results on 12 signaling pathways from the NetPath database indicate that identifying the strongest path is helpful for pathway reconstruction. Moreover, since the stored interactions in different databases may vary, simultaneous search of multiple databases is necessary. Among the available Cytoscape apps, the most similar app to ours in terms of functionality is PathLinker, which is the state-of-the-art algorithm in pathway reconstruction [4, 5]. Therefore, we only compare our application with PathLinker. In summary, our contribution is an application (StrongestPath) that provides easy access to multiple public large databases, generating output in a short time, and addressing three key challenges, all in one platform with a user-friendly graphical interface.

## Implementation

We designed StrongestPath with four main panels including *Select Databases*, *Strongest Path*, *Expand* and *Regulatory Path*. In the following, we describe each panel separately.

### Select databases

We developed StrongestPath in Java, along with R scripts to preprocess the required databases. We used the NCBI [7] and the UniProt [8] databases to build the built-in protein-coding genes nomenclature databases, which allows us to use any of 11 different gene or protein accession numbers including Entrez Gene ID, Official gene symbol, Aliases, Uniprot Gene ID, Ensembl (gene, transcript and peptide), RefSeq (peptide and mRNA), Reactome ID, and STRING ID. We also supplied the application with some PPI, signaling, and regulatory networks from public databases including STRING [9], HitPredict [10], HIPPIE [11] (only for human), KEGG [12], Reactome [13] and TRRUST [6]. Currently, both human and mouse species are supported in the application.

Once the user starts the application, if the internet connection is available, the list of supporting species by the application will be updated and the user can easily and quickly access all available databases for the selected species by only clicking on the *Download/Update Databases* button. Since the network data in public databases are often very large, we removed any non-essential information from the network data, and then converted the gene accession identifiers in the network data to their line numbers in the built-in annotation file and produced a network data with a smaller size compared to the original one. Currently, the downloaded data can be stored on a hard disk drive with less than 1GB free space and the application can be used later without any dependence on internet connection.

Furthermore, users can use the application with their own data including the annotation file and the network file. The annotation file is a tab-separated file containing different types of identifiers for the network nodes. In this file, each row refers to a specific node of the network and each column represents a list of different identifier types that must be separated by comma. Only the first column of the annotation file, which is used to label nodes, is required. Any additional column is optional. The network file is a tab-separated file containing three columns, in which each interaction is reported in one row and the columns refer to the source node, the target node, and the confidence score (i.e. a probability value between 0 and 1) respectively. For calling each node in the network file, all different accession identifiers given in the annotation file can be used.

**Fig. 1:**
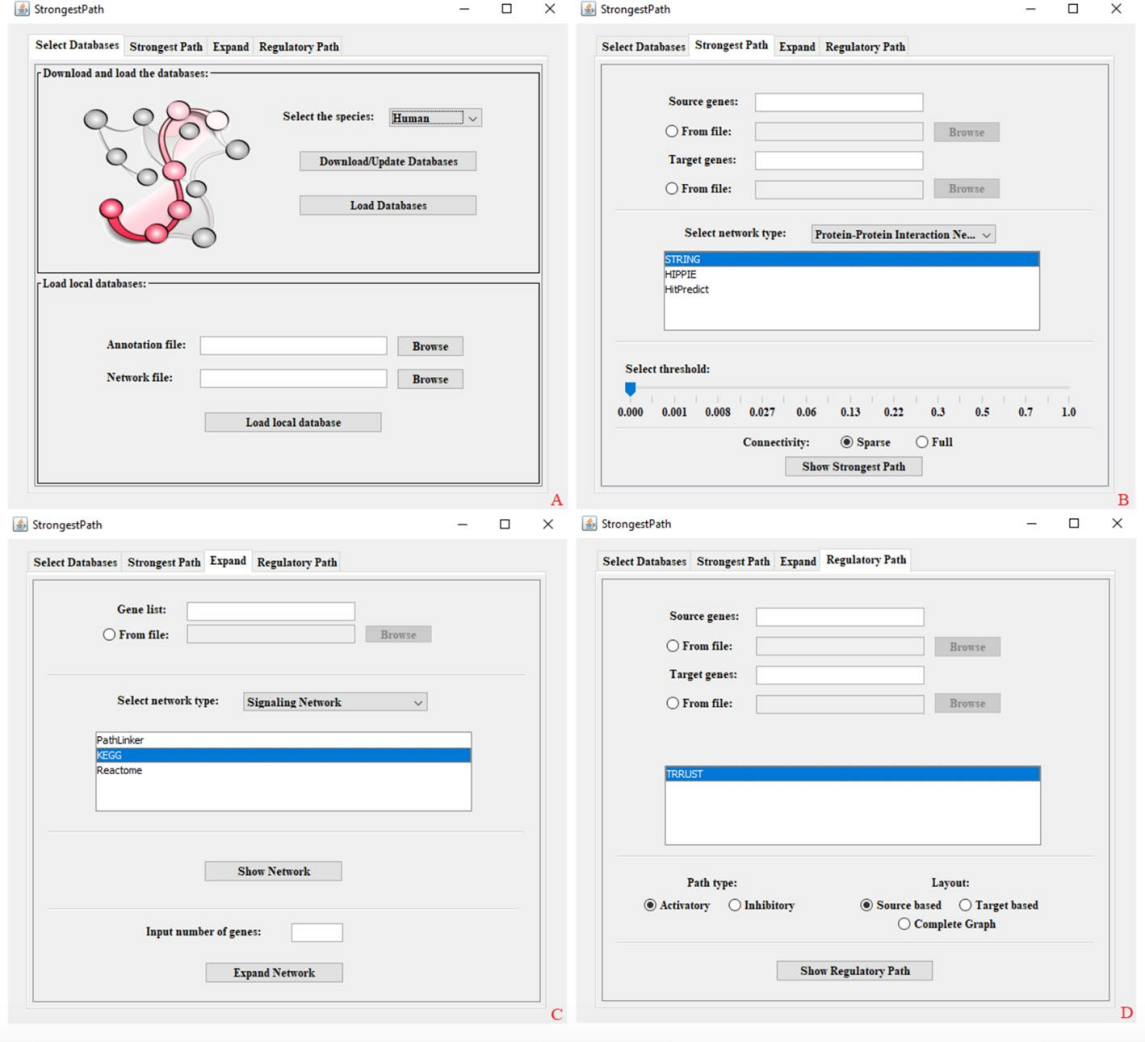
A view of StrongestPath with four panels: **a** Select Databases, **b** Strongest Path, **c** Expand and **d** Regulatory Path

As mentioned earlier, StrongestPath implements different scenarios in three distinct panels of *Strongest Path*, *Expand,* and *Regulatory Path*. In each run of the application, the built-in databases of the selected species or the user provided data should be loaded by pressing the *Loading Databases* button in the *Select Databases* panel (See Fig.1).

### Strongest path

We used interaction confidence scores for assigning weights between 0 and 1 to each edge of the PPI or signaling network. Given two sets of source and target proteins, the goal is to identify the strongest path connecting at least one source protein to at least one target protein. The number of possible pathways of any length between sources and targets can be extremely high, and finding a short pathway with highly confident interactions is not straightforward. Assuming confidence scores as the probability of interactions, we define the strongest path as the most probable chain of interactions, i.e. the path with the maximum edge weights product. In different networks, the strongest path could have different interpretations. While the strongest path can represent the most likely chain of interactions between two groups of proteins, it also represents a linear signaling pathway while the given graph is a signaling network [14, 15].

It is easy to show that identifying the path between two nodes of a general graph with maximum edge weights product is NP-Complete. This can be done by reducing the Hamiltonian Path problem, which is an NP-Complete problem, to it. By assigning a constant weight 2 to every edge, the problem would be equivalent to the Hamiltonian Path, which is also NP-Complete. However, we made a “dual” graph with the same set of edges as the original one but with modified weights, using it the exact solution can be found in polynomial time. The only requirement is that all original weights should be real numbers between 0 and 1. This requirement is met when the original weights are interaction probabilities.

Consider a weighted directed or undirected graph *G* with all edge weights as positive real numbers not greater than 1, that we call the “primal” graph. Two disjoint subsets of nodes *A* and *B* are also given from the nodes of *G* as source and target nodes, respectively. The goal is to a path $y(A,B)$ from the set of all possible paths *S* from any node in *A* to any node in *B with* the maximum path weight:

$$y(A,B)=\mathrm{arg}{max}_{\pi \in S}w(\pi )$$

where the weight $w(\pi (a,b))$ of a path $\pi (a,b)$ between nodes $a$ and $b$ to is the product of the weights of the path edgesh. If there are two paths with the same path weight, the path with less number of edges is considered the strongest path. In our method we use a constant penalty factor *D* to penalize long paths. *D* is a hyperparameter of our method that controls the importance of length of the strongest path. We create the dual graph by changing the weight of each interaction (edge) *e* to $W(e)=-(logD+logP(e))$. For all $0<P(e)\le 1$, where *e* is any edge in the graph, and *D*=0.95, the $-(logD+logP(e))$ is always a positive bounded real number. Hence, we can apply a normal shortest-paths algorithm (i.e. Dijkstra’s algorithm) on the dual graph to find the strongest path $y(A,B)$. In order to find the strongest path between two groups of nodes, we add one super-source and one super-sink to the network, connect one group to the super-source and the other group to the super-sink. This way we reduce the problem of multiple-source multiple-sink shortest path to a single-source single-sink shortest path problem.

We also modified the algorithm to be able to find sub-optimal strongest paths (i.e. paths with slightly less probability product than the maximum). For a given positive real value $\epsilon$, we define $\epsilon$-strongest path between A and B as follows:

$${X}_{\epsilon }(A,B)=\{x|x\in S,w(x)\le w(y(A,B))+\epsilon \}$$

where $w(y(A,B))$ is the length of the optimum shortest path in the dual graph. Since there are graphs where the number of A-to-B paths can be exponential in $(1+\epsilon )$, finding all paths of ${X}_{\epsilon }$ would be time consuming. However, finding the intermediate proteins that play an important role in the chain of interactions between *A* and *B* is suitable for most applications. Hence, we define ${V}_{\epsilon }$ as the set of nodes which are seen in at least one $\epsilon$-strongest path. To find ${V}_{\epsilon }$, we use the dual graph and for every node *v*, we define $a(v)$ as the weight of the shortest path from any node of *A* to *v*, and similarly $b(v)$ is the weight of the shortest path from *v* to any node in *B*. Only nodes with $a(v)+b(v)\le w(y(a,b))+\epsilon$ are inserted into the set ${V}_{\epsilon }$, and the induced sub-graph of ${V}_{\epsilon }$ will be displayed in the output graph**.** For better visualization of the result, the Breadth First Search (BFS) algorithm is employed to compute the distance of each node from *A*. The color and position of the nodes are then assigned accordingly.

Using the *Strongest Path* panel in the application, the user can find the strongest path connecting at least one source protein to one target protein, as described above. A comma-separated list of source genes and target genes can be given as input to the application by entering any accession gene identifier supported in the application. As seen in Fig. 1, a list of genes can also be given to the application via a text file containing one line per gene. By choosing a network type from one of the default types, a list of networks of that type, supported in the application, is displayed for selection. The *Show Strongest Path* button searches for the strongest path between the source and target nodes in the selected networks, and visualizes the output of each selected network in Cytoscape as a separate network. Before any search, at least one source gene, one target gene and one network must be selected by the user. As seen in Fig. 1, a slider is provided at the bottom of the panel. The user can find the sub-optimal strongest paths between source and target nodes, as defined above, by increasing the threshold value determined by the slider. When increasing the threshold parameter, the number of strongest paths increases exponentially and the output graph will be dense. By selecting the sparse option, all the proteins which are seen in at least one strongest path are identified, and the corresponding paths will be displayed. This feature saves run time and makes the output network sparse.

### Expand

In this panel, a list of input proteins is given to the application and the application returns a network containing input proteins and their connections in the selected background network at the first step. When giving a positive integer *n is given as input*, the network is expanded by adding *n* proteins whose total weight of interactions with the proteins in the network has the highest value. A list of input genes can be entered into the application directly or by providing a text file. The input format in the whole application is similar to what was mentioned earlier. After choosing the network type, a list of loaded networks in the application is given for selection as indicated in Fig. 1. The *Show Network* button searches for a network of interactions among input genes based on the interactions reported in the selected databases. If the user selects more than one database, the network associated with each database is shown in Cytoscape separately. Each of these networks can be expanded by a number of close neighbours by clicking the *Expand Network* button.

### Regulatory path

To answer whether there is any activating or inhibitory path between source genes, encoding transcription factors, and target genes, the user can use the *Regulatory Path* panel (Fig. 1). In this panel, the application searches the TRRUST database [6], an experimentally validated database containing human TF-target links with mode of regulation information, to identify regulatory paths. We use the BFS algorithm to compute the shortest path between any pair of source and target genes. In this case, we define the shortest path as a path connecting source and target genes with the minimum number of links. If the path is available, weights of + 1 and − 1 are assigned to activating and inhibitory links of the path, respectively, based on the information about mode of regulation in TRRUST [6]. In the simplest case, there are two situations for each regulatory path, when a change of level of source gene causes the change of level of target gene. If the presence of source gene implies the presence of target gene and conversely the absence of source gene implies the absence of target gene, the path is called activating path. The opposite situation corresponds to the inhibitory path. Accordingly, if the edge weights product is + 1, the shortest path is defined as an activating path, otherwise the path is defined as an inhibitory path.

In this panel, source and target genes can be given to the application as input, similar to the other panels. Currently, only TRRUST database is available in the application for finding regulatory paths. After pressing the *Show Regulatory Path* button, the application computes the regulatory paths with the selected mode of regulation between source and target genes, as defined above, based on the reported information in the TRRUST database.

## Results

### Strongest path

To demonstrate the effectiveness of StrongestPath, we used 12 signaling pathways provided in the NetPath [16] database. The signaling receptors and transcription factors of each pathway were identified using the NetSlim [17] and the MSigDB [18] databases respectively. For each pathway, the receptors and TFs were given as source and target genes to the application, respectively, and then we used the application to find the strongest path(s) between sources and targets in a background network. Since two types of networks including signaling networks and protein interaction networks can be selected in the application as a background network, we selected KEGG and STRING networks in separate runs. The current version of KEGG network for human species, derived from the aggregation of all KEGG signaling pathways, includes 6326 proteins and 61980 interactions. Since KEGG is a curated database, the probability score of all network interactions were considered equal to 1. The STRING network for the human species is a very large protein interaction network consisting of 18725 proteins and more than 5 million interactions, and all links were weighted by a confidence score between 0 and 1. Although the background networks, specially the STRING network, are very large, the application is able to find the strongest path(s) between multiple source and target genes in a few seconds. In addition to the strongest path(s), we also identified sub-optimal strongest path(s) between source and target genes in the STRING network by increasing the threshold parameter three times. Since all links of the KEGG have the same probability score, the number of links in the path determines the weight of the path and increasing the threshold parameter. In most cases, it leads to the addition of a large number of genes to the detected sub-network. Therefore, we used the application to identify only the strongest path(s) between source and target genes in the KEGG network containing at least one gene in the middle of the path. To assess the performance of the application, for each pathway, we investigated how many of the genes found by the application in each run were already known as pathway genes in the NetPath database. The obtained results are given in Tables 1 and 2.

**Table 1:** Details of identified strongest path(s) by the application using the KEGG background network

Pathway	# found genes	# pathway genes	FDR-corrected *p* value	List of found pathway genes
BDNF	4	2	$3.54\times {10}^{-3}$	RAC1, TRAF1
EGFR	11	10	$7.99\times {10}^{-10}$	CBL, CBLB, CBLC, ERBB2, HRAS, JAK1, KRAS, MAPK1, MAPK3, SRC
Hedgehog	10	5	$1.20\times {10}^{-8}$	ARBB2, PRKACA, SMO, STK36, SUFU
IL-1	4	3	$1.35\times {10}^{-5}$	MYD88, TAB1, TRAF6
IL-2	5	3	$5.05\times {10}^{-5}$	JAK1, JAK2, JAK3
IL-3	4	3	$2.88\times {10}^{-5}$	JAK1, JAK2, TYK2
IL-6	5	4	$6.37\times {10}^{-7}$	JAK1, JAK2, TYK2, SOCS3
IL-7	5	2	$3.06\times {10}^{-4}$	JAK1, JAK3
TCR	26	15	$5.28\times {10}^{-12}$	CHUK, FYN, IKBKB, IKBKG, MAP3K14, MAP3K7, MAPK1, MAPK11, MAPK12, MAPK13, MAPK14, MAPK3, MAPK9, MAP3K1, MAPK8
TGFB	31	15	$1.74\times {10}^{-11}$	MAPK1, MAPK3, PPP2CA, PPP2CB, PPP2R1A, PPP2R1B, RHOA, RPCK1, ROCK2, SMAD1, AKT1, MAPK8, RAF1, WWTR1, YAP1
TNF-alpha	10	10	$5.28\times {10}^{-12}$	CASPB, IKBKB, MAP3K1, MAPK10, MAPK8, MAPK9, TAB1, TRADD, TRAF2, TRAF6
WNT	48	25	$5.32\times {10}^{-22}$	CREBBP, CSNK2A1, CSNK2A2, CSNK2B, DVL1, DVL2, DVL3, EP300, GSK3B, MAPK10, MAPK8, MAPK9, PLCB1, PLCB2, PLCB3, PLCB4, PPP3CA, PPP3CB, PPP3CC, PPP3R1, PPP3R2, AKT1, MAPK1, MAPK3, SRC

**Table 2:** Details of identified strongest path(s) and sub-optimal strongest path(s) by the application using the STRING background network in four separate runs

Pathway	Iteration 1			Iteration 2	Iteration 3	Iteration 4
	#pathway genes/#found genes	FDR-corrected *p* value	List of found pathway genes	#pathway genes/#found genes	#pathway genes/#found genes	#pathway genes/#found genes
BDNF	3/4	$2.31\times {10}^{-6}$	GRB2, NGF, SHC1	7/12	12/21	14/28
EGFR	6/8	$1.85\times {10}^{-8}$	AKT1, CBL, GRB2, HRAS, JAK2, PIK3CA	9/22	12/49	19/85
Hedgehog	5/5	$2.34\times {10}^{-13}$	DHH, HHIP, SHH, SMO, SUFU	7/17	7/28	7/46
IL-1	4/5	$3.36\times {10}^{-9}$	IKBKB, MAP3K7, TAB2, TRAF6	7/10	7/19	9/38
IL-2	2/6	$3.61\times {10}^{-4}$	IL2, JAK2	6/15	8/25	8/31
IL-3	0/6	$1$		2/11	2/22	3/43
IL-6	4/8	$7.17\times {10}^{-8}$	AKT1, IL6, JAK1, JAK2	5/13	5/27	5/45
IL-7	1/5	$8.12\times {10}^{-3}$	JAK3	4/12	4/15	4/33
TCR	3/4	$2.32\times {10}^{-5}$	IKBKB, MAP3K7, TNF	4/7	4/9	5/20
TGFB	8/8	$2.01\times {10}^{-14}$	TGFB3, ZFYVE9, AKT1, AXIN1, FKBP1A, GSK3B, MTOR, NEDD4L	9/11	13/37	16/150
TNF-alpha	3/3	$8.82\times {10}^{-6}$	IKBKB, IKBKG, TNF	7/7	11/20	12/40
WNT	3/3	$2.31\times {10}^{-6}$	APC, AXIN1, WNT1	7/9	10/18	14/37

For all pathways, as seen in Table 1, where we used the KEGG network to find the strongest path(s) between receptors and TFs of each pathway, more than 50% of genes found by the application were already known as pathway genes in the NetPath database. Also when the STRING network was used, as reported in the first column of Table 2 (i.e. Iteration 1), approximately 80% of genes in the identified strongest path(s) (and 100% for some pathways) were reported to be pathway genes in the NetPath database. As seen in the next columns of Table 2 (i.e. Iterations 2, 3 and 4), by increasing the threshold parameter and identifying sub-optimal strongest path(s), this amount will decrease.

Our results demonstrate that the application can be used to identify which genes can play a role in the middle of the pathway by finding the strongest path(s) in the signaling network like KEGG, or in the protein interaction network such as STRING. Since the networks are available in the application for both human and mouse species, StrongestPath can be used more easily compared to similar Cytoscape apps like PathLinker. More species can be added to the application in the future without any new installation, meanwhile, the application can be used for other species by giving the annotation and the network files to the app manually.

In both Tables 1 and 2, the number of found genes refers to the number of genes that were found by the application in the middle of the strongest paths. Also, the number of pathway genes is the number of genes from the set of found genes that were known to belong to a specific pathway. For each pathway, the p-value was calculated by hypergeometric distribution to quantitatively assess the significance of the overlap between the application output and the pathway genes. We used the “phyper” function in R to calculate the *P* values, and the false-discovery rate (FDR) was used to account for multiple testing. When the FDR-corrected p-value is close to zero, it means that most of the genes in the strongest path(s) have already been identified as genes of a given pathway, and it is unlikely that this happens by chance.

According to the obtained results on the above signaling pathways, analyzing the strongest path(s) between source and target genes by considering different PPI or signaling networks as a background network can detect different sets of proteins in the middle of the path, with little in common. Therefore, researchers can easily use StrongestPath with multiple PPI or signaling networks provided in the application to find the proteins that can play a significant role in the middle of the pathway between source and target proteins.

### Expand

**Fig. 2:**
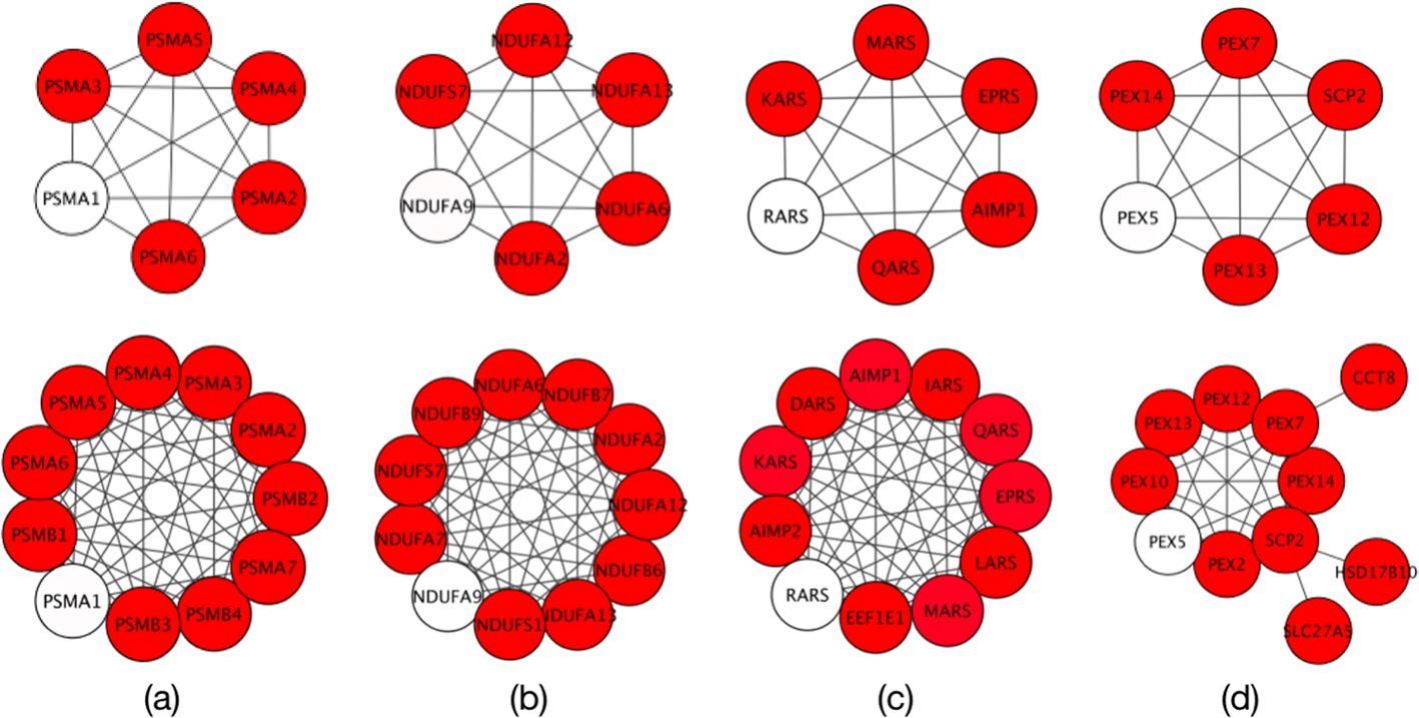
An example of using the Expand panel to identify proteins of four protein complexes: **a** proteasome, **b** respiratory electron transport, **c** aminoacyl-tRNA biosynthesis, and **d** peroxisome

As mentioned earlier, the Expand panel can be used to identify unknown elements of a protein complex, biological process or core regulatory circuitry. To demonstrate the functionality of the application in the Expand panel, four different protein complexes associated with the proteasome, respiratory electron transport, aminoacyl-tRNA biosynthesis and peroxisome pathways have been selected and one protein from each complex, respectively *PSMA1*, *NDUFA9*, *RARS* and *PEX5*, have been given to the application in separate runs. At each run, we expanded the network twice, and each time, five proteins which have the strongest interactions with the existing proteins were identified from the STRING database and added to the network. As seen in Fig. 2, all proteins added to each network belong to the same protein complex and are involved in the similar signaling pathway.

### Regulatory path

**Fig. 3:**
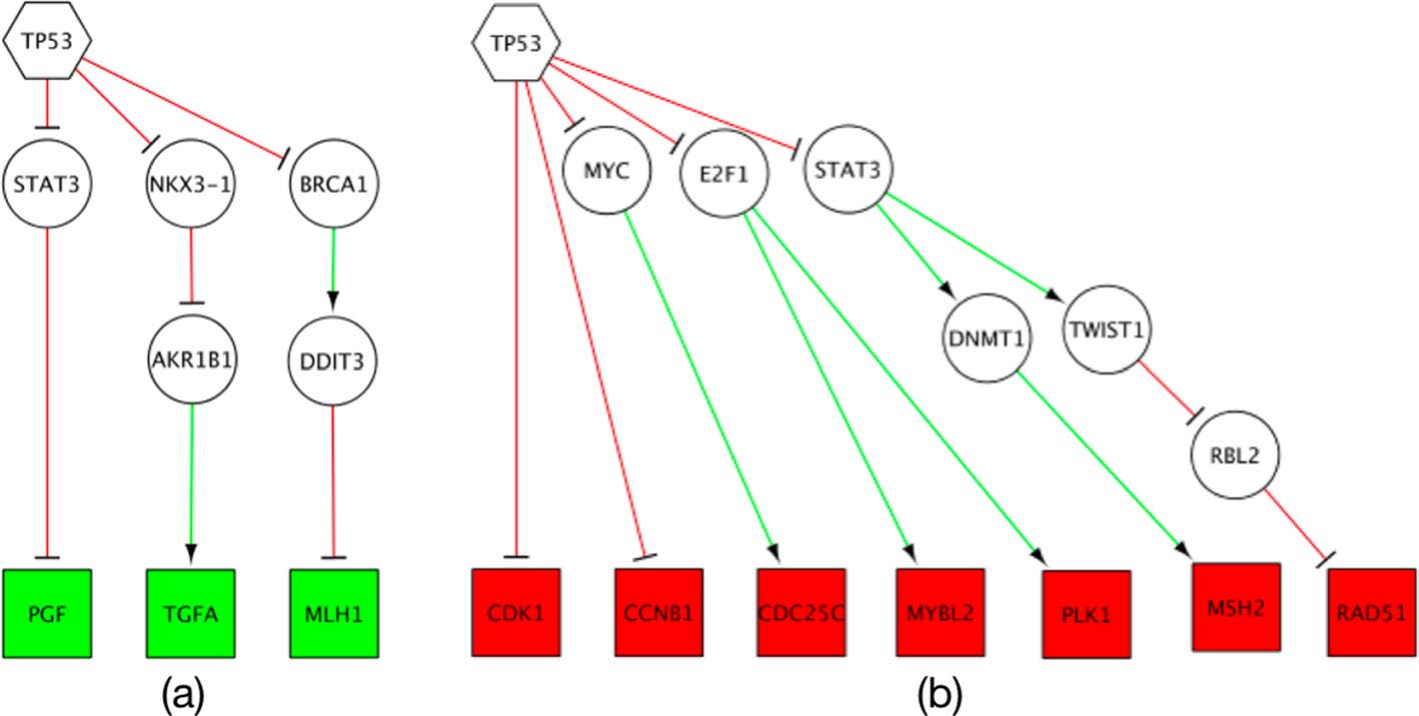
An example of using the Regulatory panel to find regulatory paths, **a** activating and **b** inhibitory, between *TP53* and a set of cell cycle arrest and DNA repair genes. Green and red colours are used for colouring activating and inhibitory links in the output. Up-regulated and down-regulated target genes are also coloured with green and red respectively.

As discussed earlier, the Regulatory panel can be used to identify both activating and inhibitory regulatory paths between source genes, encoding transcription factors, and target genes. Here, we provide an example to illustrate the biological utility of this panel. The *TP53* gene encodes a TF which acts as a tumour suppressor. Target genes of *p53* function in multiple biological processes, including cell cycle arrest and DNA repair. Suppose we have a list of cell cycle genes, including *CDK1*, *CCNB1*, *CDC25C*, *MYBL2*, *PLK1*, *PGF* and *TGFA*, and a set of DNA repair genes, including *RAD51*, *MSH2* and MLH1, and we want to identify which of the genes are directly or indirectly targeted by the p53. Given the *TP53* gene as source gene and the cell cycle genes and the DNA repair genes as target genes, the Regulatory panel retrieves any inhibitory and activating path between the source TF and the target genes. Interestingly the results of the application, as provided in Fig. 3, are in accordance with the data reported in [19]. In Fig. 3, the target genes which coloured with green including *PGF*, *TGFA* and *MLH1* were experimentally confirmed to be activated by the p53 gene in [19]. Moreover, it is confirmed that the p53 gene also inhibits cell cycle genes such as *CDK1*, *CCNB1*, *CDC25C*, *MYBL2* and *PLK1*. Also, some DNA repair genes including *MSH2* and *RAD51* were reported to be indirectly repressed by p53 gene [19].

### Comparison with PathLinker

In this section, we only compare StrongestPath with PathLinker, which is a Cytoscape application with better accuracy compared to the others for pathway reconstruction, as stated in [4, 5]. The PathLinker uses A*-augmented Yen’s Algorithm to find the $k$-shortest paths from $A$ to $B$, as defined above. Given a network with $n$ nodes and $m$ links, PathLinker runs in $O(nk(m+nlogn))$, and the runtime of the algorithm linearly scales with the value of $k$. StrongestPath executes the Dijkstra’s algorithm only twice, once on the primal graph and once on the dual graph. So, regardless of the value of $\epsilon$, StrongestPath runs in $O(m+nlogn)$. Therefore, StrongestPath runs in $O(nk)$ times faster than PathLinker.

**Fig. 4:**
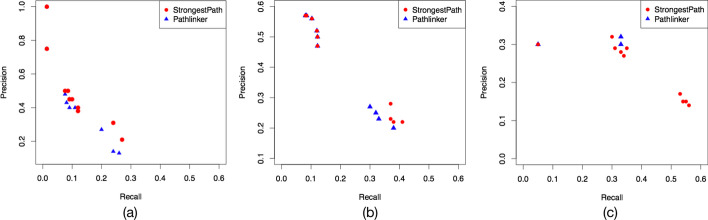
The precision-recall plots of StrongestPath and PathLinker for three signaling pathways including: **a** $WNT$, **b** $TGF\beta$, and **c** $TNF\alpha$.

To compare both applications in terms of precision and recall measures, the applications were used for the reconstruction of the three signaling pathways of $WNT$, $TGF\beta$, and $TNF\alpha$. For each pathway, the sets of source and target nodes, similar to what was given in [5], were used in both applications as input sets and we used the PathLinker network as the background network. The PathLinker network is a weighted network, containing 12,046 nodes and 152,094 directed links, constructed by the authors of PathLinker application from many protein–protein interaction and signaling pathway databases [4]. We executed StrongestPath and Pathlinker with ten different values of $\epsilon$ and $k$, respectively. For each value of $\epsilon$, the corresponding value for $k$ was identified. As expected, since the same idea is used in both applications, the applications output is similar in finding the strongest paths. However, each application uses a different algorithm with a different run-time complexity for finding the genes involved in the sub-optimal strongest paths, i.e. the $\epsilon$-strongest paths. As mentioned earlier, a list of genes involved in each pathway was identified using the NetPath database. Precision is the fraction of genes involved in a pathway among the identified genes in the $\epsilon$-strongest paths. Recall is the fraction of pathway genes that were retrieved in the $\epsilon$-strongest paths. For each pathway, we calculated precision and recall measures from the outputs of both applications using increasing values of k and $\epsilon$. As seen in Fig. 4, StrongestPath performs better than PathLinker for the large values of the parameter $k$ and $\epsilon$.

In terms of run time, each run of StrongestPath took about one second, while PathLinker takes more time especially for large values of $k$, as reported in [5] (see Table 3). Moreover, if we select the Include tied paths parameter in PathLinker, there are a huge number of paths with the product of edge weights similar to the *k*-th strongest path. So, due to the time complexity of PathLinker, finding all of these paths is not computationally feasible. For example, using the $TNF\alpha$ pathway, when we changed the value of $k$ to 10,000, PathLinker was unable to identify all paths with the product of edge weights similar to the *k*-th strongest path even after 30 minutes.

**Table 3:** Time comparison between StrongestPath and PathLinker using three signaling pathways of $WNT$, $TGF\beta$, and $TNF\alpha$ for increasing values of *k*.

Pathway	WNT pathway		TGFβ pathway		TNFα pathway	
# of sources	14		5		4	
# of targets	14		77		44	

*k*	PathLinker	StrongestPath	PathLinker	StrongestPath	PathLinker	StrongestPath
100	5.2	1	4.7	1	3.3	1
1000	13.8	1	10.5	1	7.7	1
10,000	144.4	1	116.8	1	86.0	1

Furthermore, StrongestPath is more applicable than PathLinker for the reasons listed below:

1. Both PathLinker and StrongestPath allow users to use their own networks. In addition, StrongestPath allows the use of large networks from public databases such as KEGG and STRING. To achieve the same goal in PathLinker, one has to load this data manually, which for large networks such as STRING, it would be impossible.
2. The users can input a list of proteins into StrongestPath via a number of different nomenclatures. So the user doesn't need to know one specific identifier of their input proteins and in most cases the ID mapping is not necessary to be done before using our application.
3. In the current version of StrongestPath, identifying regulatory paths (Activating/ Inhibitory) between transcription factors and target genes can also be done using the TRRUST database.

## Conclusions

In summary, StrongestPath is a Cytoscape application for protein–protein interaction and signaling network analysis. It allows the user to search for strongest path(s) or sub-optimal strongest path(s) in a PPI or signaling networks for pathway reconstruction, to create and expand network of interactions among a list of proteins, and to explore activating or inhibitory regulatory paths between TFs and target genes in a regulatory network. Easy access to some public large databases of human and mouse species and a user-friendly graphical interface make this application more convenient for the users. Moreover, the application can be easily expanded for supporting more species and also networks from more public databases in the future without having to install another version of the application only with an internet connection.

## Data Availability

All datasets generated and analysed during the current study are available in the GitHub repository, [http://github.com/zmousavian/StrongestPath].

## Availability and requirements

**Project name:** StrongestPath: a Cytoscape application for protein–protein interaction analysis.

**Version:** 2.0.

**Operating systems:** Platform independent.

**Programming language:** Java.

**Software requirements:** Cytoscape 3.0 (http://www.cytoscape.org/).

**License:** GPL.

**Any restrictions to use by non-academics:** none.
